# Omega-3 Polyunsaturated Fatty Acids, Gut Microbiota, Microbial Metabolites, and Risk of Colorectal Adenomas

**DOI:** 10.3390/cancers14184443

**Published:** 2022-09-13

**Authors:** Tengteng Wang, Nicole M. Brown, Amber N. McCoy, Robert S. Sandler, Temitope O. Keku

**Affiliations:** 1 Channing Division of Network Medicine, Department of Medicine, Brigham and Women’s Hospital and Harvard Medical School, Boston, MA 02115, USA; 2 Center for Gastrointestinal Disease and Biology, University of North Carolina, Chapel Hill, NC 27599, USA; 3 Department of Medicine, University of North Carolina at Chapel Hill, Chapel Hill, NC 27599, USA; 4 Department of Nutrition, University of North Carolina at Chapel Hill, Chapel Hill, NC 27599, USA

**Keywords:** ω-3 polyunsaturated fatty acid, mucosal-adherent microbiota, bile acids, metabolite, colorectal adenomas

## Abstract

**Simple Summary:**

Dietary omega-3 polyunsaturated fatty acids (ω-3 PUFAs) may protect against colorectal adenoma development, and the gut microbiota and microbial metabolites (particularly bile acids) are important in dietary fat metabolism. We aimed to evaluate the impacts of ω-3 PUFAs, gut microbiota, and bile acids (BAs) on colorectal adenoma occurrence in a case–control study (*n* = 435) with 16s rRNA sequencing and global metabolomics (subset *n* = 50) measurements. We observed that ω-3 PUFA intake was associated with an 11–55% risk reduction in developing colorectal adenoma, and the association was modified by the gut bacterial evenness level. We also found that three specific gut bacteria and four BAs metabolites that were measured in normal colonic mucosa tissue were positively associated with colorectal adenomas. These findings provide important insights and imply that the improvement of ω-3 PUFA intake and/or alterations in the gut microbial environment may become a potential risk reduction strategy for colorectal cancer prevention.

**Abstract:**

Omega-3 polyunsaturated fatty acids (ω-3 PUFAs) are thought to protect against colorectal adenoma (CRA) development. We aimed to further understand the underlying mechanisms by examining the relationships between ω-3 PUFAs and the gut microbiota on CRAs. We assessed the mucosal microbiota via bacterial 16S rRNA sequencing among 217 CRA cases and 218 controls who completed PUFA intake questionnaires. The overall microbial composition was assessed by α-diversity measurements (diversity, richness, and evenness). Global metabolomics was conducted using a random subset of case–control pairs (*n* = 50). We compared microbiota and metabolite signatures between cases and controls according to fold change (FC). Odds ratios (OR) and confidence intervals (CI) were estimated from logistic regression for associations of ω-3 PUFAs and the microbiota with CRAs. We observed an inverse association between overall ω-3 PUFA intake and CRAs, especially for short-chain ω -3 PUFAs (OR = 0.45, 95% CI: 0.21, 0.97). Such inverse associations were modified by bacterial evenness (*p*_-interaction_ = 0.03). Participants with higher levels (FC > 2) of bile acid-relevant metabolites were more likely to have CRAs than the controls, and the correlation between bile acids and bacterial diversity differed by case–control status. Our findings suggest that ω-3 PUFAs are inversely associated with CRA development, and the association may be modified by gut microbiota profiles.

## 1. Introduction

The public health burden of colorectal cancer is considerable. Approximately 151,030 new cases of colorectal cancer are expected to occur among the US population in 2022 [1]. It has been widely accepted that the majority of colorectal malignancies develop from colorectal adenomas in a morphological progression termed the adenoma–carcinoma sequence [2]. Therefore, identifying the causes of colorectal adenomas has significant public health ramifications for preventing colorectal cancer.

Accumulating laboratory studies support the hypothesis that ω-3 PUFAs may protect against colorectal carcinogenesis, mainly through inhibiting inflammation and cell proliferation processes [3,4]. There are different subtypes of ω-3 PUFAs that are defined by the length of carbon atoms. For example, a-linolenic acid (ALA) contains 18 carbon atoms and is therefore considered a short-chain (SC) ω-3 PUFA, whereas eicosapentaenoic acid (EPA) and docosahexaenoic acid (DHA) contain 20 or more carbon atoms and are termed long-chain (LC) ω-3 PUFAs. Given their different chemical structures, the nutritional characteristics of SC and LC ω-3 PUFAs also differ [5]. The epidemiologic data on SC and LC ω-3 PUFAs in relation to colon carcinogenesis are inconclusive, and the underlying mechanisms are poorly understood. A recent meta-analysis of 14 prospective studies failed to show conclusive evidence of the protective effect of either SC or LC ω-3 PUFAs on colorectal cancer risk [6]. Furthermore, although observational studies support a protective effect of EPA, the first randomized trial of EPA on colorectal adenoma chemoprevention found a null effect on risk reduction [7]. These data suggest that the effect of SC and LC ω-3 PUFAs on colon adenoma and cancer risk merits further investigation [6], especially for identifying subgroups of individuals who may benefit the most from consuming different subtypes of ω-3 PUFA.

The gut microbiota may play an important role in explaining the underlying mechanisms of ω-3 PUFAs on colon carcinogenesis [8] because the gut microbiota is involved in the metabolism of dietary fat in the intestine through the transformation of bile acids (BAs) [9] and is also involved in colorectal carcinogenesis [10,11,12,13]. There are several approaches to characterizing the gut microbiota in relation to disease. On a comprehensive level, the overall microbiota composition may be described by traditional measures such as diversity (the number microbes present), richness (the number of taxa), and evenness (the distribution of taxa) [14]. At the individual level, the abundance of specific bacterial taxa can be evaluated relative to specific disease conditions. In our previous studies, we showed increased bacterial richness and enrichment of *Fusobacterium* species in adenoma cases compared to in non-adenoma controls [10,11]. Other studies examined how gut microbiota and colorectal adenomas/cancer have reported similar findings of bacterial dysbiosis [15,16,17], but they had a small sample size and were often limited to fecal microbiota and/or a small number of bacterial species being analyzed.

Previous studies [18,19] have shown that a high-fat diet (e.g., saturated fatty acids) increases the hepatic synthesis of primary BAs; hence, the concentrations of secondary BAs are increased after bacterial transformation [20,21]. Both primary and secondary BAs are known to generate reactive oxygen species (ROS) and reactive nitrogen species (RNS), which have been implicated in carcinogenesis in different regions of the intestinal tract [21]. Thus, it is becoming increasingly clear that the complex interactions between dietary factors, the resident gut microbiota, and their metabolic products, such as secondary BAs, have the potential to influence the development of colorectal adenoma and cancer [21]. Therefore, we hypothesized that the inconsistent results for ω -3 PUFAs in relation to colorectal adenomas could be due to changes in the gut microbiota and their metabolites.

Currently, no large human studies have assessed the potential interactions of ω-3 PUFAs, gut bacteria, and BA on developing colorectal adenomas. In this case–control study with a relatively large sample size and mucosal tissue-based microbial metabolites, we first aimed to examine the associations of ω-3 PUFA intake and adherent gut microbiota (and their interaction) in relation to colorectal adenomas. Furthermore, we assessed the associations between BAs, gut microbiota, and adenomas in the subset of samples with metabolomics data.

## 2. Methods

### 2.1. Study Population

Our analytical population was identified from the Diet and Health Study V (DHS V), which included participants (age ≥ 30 years old) who had undergone screening colonoscopies at the main hospitals of the University of North Carolina (Chapel Hill, NC) between August 2008 and March 2010. Those who provided informed consent, agreed to take part in a phone interview, consented to provide rectal biopsies during the procedure, and/or agreed to have blood drawn were considered to be eligible for this study. Participants who were less than 30 years old; unable to provide informed consent; had polyposis (>100 polyps), prior colon resection or cancer, colitis, prior colon adenomas, familial polyposis syndrome, inadequate preoperative, or incomplete examination (cecum not reached); and who had used antibiotics within the previous three months were excluded [22]. All elevated lesions were excised or biopsied during the colonoscopy and were sent directly for pathohistological review. The research pathologist evaluated the histology of each colon polyp and categorized each polyp according to accepted pathologic standards [22]. Cases were defined as participants with one or more adenomatous polyps. Those who had no adenomatous polyps were classified as controls. In total, 217 cases and 218 controls were eligible for this analysis. This study was approved by the institutional review board (IRB) at the School of Medicine, University of North Carolina.

### 2.2. Data Collection

#### 2.2.1. Dietary and Lifestyle Exposures

Information about demographic characteristics, education, family history, medical history, nonsteroidal anti-inflammatory drug (NSAID) use, smoking, and other lifestyle exposures was collected by telephone interview using a well-structured questionnaire within 12 weeks of the colonoscopy. The interviewer did not know the results of the colonoscopy. Dietary intake during the year prior to the colonoscopy was assessed using the National Cancer Institute quantitative food frequency questionnaire [23]. Intake of total ω-3 PUFAs was estimated as the sum ALA, stearidonic acid (SDA), EPA, DHA, and docosapentaenoic acid (DPA). To reflect the measures of ω-3 PUFA subtypes, LC ω-3 PUFA intake was computed to reflect the sum of EPA, DHA, and DPA. ALA and SDA were summed to represent the intake of SC ω-3 PUFAs. Subjects were also asked if they had made changes to their diet based on symptoms or for health reasons, when these changes were made, and why. DHS research staff weighed all subjects and measured their height and waist and hip circumferences at the time of the colonoscopy [11].

#### 2.2.2. Biopsy Sample Collection

Biopsy collection was carried out as previously described [11,24]. Preparation for the colonoscopy included a 12 h fast and bowel cleansing with polyethylene glycol. Normal rectal mucosal biopsies were taken from each patient immediately following the insertion of the scope at about 10 to 12 cm from the anal margin to minimize disrupting the mucosa [11]. The number, location, and size of the adenomas were recorded during the collection, but we only selected biopsies from normal mucosa for all the subjects, as the adenomas were used for further clinical diagnosis [11,24,25]. The normal mucosal biopsies were rinsed onsite in sterile phosphate-buffered saline, were then snap-frozen in liquid nitrogen, and were later kept at −80 °C until bacterial DNA extraction [11,24].

#### 2.2.3. DNA Extraction and Bacterial 16S rRNA Sequencing

Bacterial genomic DNA was extracted according to standard procedure [24]. Briefly stated, two normal mucosal biopsies (10–20 mg each) were taken from each subject and were incubated with lysozyme (30 mg/mL; Sigma, St. Louis, MO, USA) for 30 min before being homogenized on a bead beater (Biospec Products Inc., Bartlesville, OK, USA) and before being extracted using the Qiagen DNeasy Blood & Tissue kit (Qiagen, Valencia, CA, USA). Aliquots of DNA samples were kept at −20 °C.

Sequencing of the microbiota was performed using a modified protocol of Fadrosh et al. [26]. First, libraries were prepared in two steps using primers to amplify the V2 region of the 16S bacterial rRNA gene. The Illumina Index barcode, adaptor, and tag sequences were used in the second PCR phase. The PCR product was examined on E-gel 96 (Life Technologies, Carlsbad, CA, USA) to ensure amplification and correct product size using the Sequal Prep Kit (Life Technologies, Carlsbad, CA, USA). All amplified samples were normalized to 25 ng/mL, and an equal volume of each sample was pooled. At the University of Maryland, the pooled product was sequenced using an Illumina MiSeQ.

The DNA extraction, PCR, and sequencing processes all included appropriate negative and positive controls for quality assurance.

We used appropriate controls during the DNA extraction, PCR, and sequencing steps, which included a Bacteria Mock Community consisting of aerobes and anaerobes, negative DNA extraction and reagent controls (i.e., water and reagents), and no template control for PCR.

#### 2.2.4. Bacterial Identification

Raw bacterial 16S sequences were quality filtered and analyzed using QIIME [27], following which the sequences were clustered into Operational Taxonomic Units (OTU) at a 97% similarity. Taxanomic assignments for the OTUs were performed by running a BLAST search using the GreenGenes database. The OTU table was used for downstream analysis to assess the microbial profiles between the cases and controls.

#### 2.2.5. Bacteria Overall Composition and Diversity Measurements: Diversity, Evenness and Richness

The Shannon–Wiener Diversity Index (H), which measures diversity, was derived using the formula H = −$\sum Pi\;\left(lnPi\right),$ where *Pi* is the proportion of each species (taxa) in the sample [11]. Richness (S) was evaluated as the number of OTUs, genera, or phyla found across 2636 sequences, where 2636 refers to the sample’s total number of sequences [11]. For each sample, 2636 sequences were randomly chosen 1000 times, and the average number of OTUs, genera, or phyla observed over 1000 permutations was estimated as richness [11]. Evenness (J) evaluates how equally the individuals are distributed among the various species/taxa, and it is calculated by J = H/Log (S), where H (diversity) is Shannon–Wiener diversity index, and S is the number of species or taxa in each sample [11].

#### 2.2.6. Metabolome Assays

Non-targeted metabolomics was performed using normal rectal tissue biopsies from a subset (due to the assay cost) of randomly selected samples (25 CRA cases and 25 controls without adenomas) using Metabolon (Metabolon, Inc. Durham, NC, USA). Following receipt, the samples were stored at −80 °C until analysis. The samples were extracted and prepared for metabolome analysis as previously described [28]. Briefly, the samples were processed via the Intact Sample Extraction (ISE) process, which uses a methanol-based method to maximize the metabolites that can be detected in very small samples. For analyses conducted using the gas chromatography–mass spectrometry (GC/MS) and liquid chromatography–tandem mass spectrometry (LC/MS/MS) platforms, the extracted samples were separated into equal portions. Several technical replication samples were made as controls from a homogeneous pool that included a small number of all research samples (“Client Matrix”). Additional quality assurance (QA) and quality control (QC) included spiking a selection of known compounds into each sample to evaluate the process and to facilitate data curation. Following this process, samples were subjected to LC/MS/MS and GC/MS, and data peaks were identified using Metabolon’s proprietary peak integration program. For each sample, metabolite annotation was performed via comparison to a library of purified compounds or recurrent unknown compounds.

### 2.3. Statistical Analysis

We used multivariable logistic regression to estimate the adjusted odds ratios (ORs) and 95% confidence intervals (CIs) for the relationship between ω-3 PUFA intake and colorectal adenomas. We categorized the participants into subgroups based on the distribution of ω-3 PUFA intake (by tertile) among the controls. Potential confounders were selected a priori based on the literature and a directed acyclic graph [29]. These confounders included age (continuous), race (white, black, and other), sex (male, female), family history (having had at least one first-degree relative with colorectal cancer or not), NSAID use (ever, never), body mass index (BMI) (<25 kg/m^2^, 25–29.9 kg/m^2^, ≥30 kg/m^2^), cigarette smoking (yes, no), total energy intake (continuous), fruit and vegetable consumption (continuous), red meat consumption (continuous), saturated fat consumption (continuous), vitamin E consumption (continuous), and overall bacterial composition diversity estimators (diversity, richness, and evenness, continuous). Backwards elimination and the change-in-estimate approach were then used to simplify the full adjustment set and to assess the extent of the missing data of covariates (removed from the model if they did not alter the main effect estimate by > 10%). Therefore, the final multivariable models for ω-3 PUFA–adenoma association were only adjusted for age, sex, race, BMI, total energy intake, saturated fat, vitamin E, and overall bacterial composition estimators.

Statistical analysis of the gut microbiota and mucosal metabolome was performed in MicrobiomeAnalyst [30] and Metaboanalyst, respectively [31]. Relative abundances of the gut microbiota were normalized via the total sum scaling method followed by log transformation and comparative analysis. Differences in the microbiota profiles between adenoma cases and the adenoma-free controls were assessed by fold changes, significance tests (RNAseq method), principal component analysis (PCA), and partial least squares discriminant analysis (PLS-DA) [31]. Fold changes were determined by dividing the relative abundance/concentration of the microbiota in the cases by the relative data in the controls.

For metabolome analysis, we used a similar approach to the one used for the microbiota. Briefly, metabolite concentrations were normalized by the total sum scaling method followed by log transformation and comparative analysis, PCA, and PLS-DA. For both microbiota and metabolome analyses, significance tests comparing the profiles of the cases and controls were adjusted for multiple comparisons using the False Discovery Rate (FDR) method [31,32]. Microbiota and metabolites with FDR-adjusted *p*-values of < 0.05 or fold change (FC) thresholds of >2 (or reversely <0.5) were considered statistically significant. Furthermore, the significant bacteria and metabolites were entered into a logistic regression model to further examine their relationship with colorectal adenomas.

For interaction analysis, the bacterial richness, evenness, diversity, and abundance of significant specific bacteria were further dichotomized based on the median among the controls, and we used the level ≤ median as the referent group. A multivariable joint effect analysis to assess the potential interaction between the gut microbiota and ω-3 PUFAs was performed by including the interaction terms of the gut microbiota composition/abundance and ω-3 PUFAs in the model. Models with and without interaction terms were compared to compute the likelihood ratio test (LRT), with α = 0.05 [33]. Given that we only had a subset of subjects with metabolomics data, we were unable to perform the formal interaction analysis because of the limited sample size. Instead, Spearman rank correlation analysis was further used to estimate the correlation coefficients of the association of gut microbiota composition/abundance in relation to BA concentration. Differential BA concentrations according to ω-3 PUFA levels and the gut microbiota were also assessed via the fold change method.

All analyses were conducted using MicrobiomeAnalyst [30], MetaboAnalyst 3.0 [31], and SAS version 9.4 (SAS institute, Cary, NC, USA).

## 3. Results

### 3.1. Study Participant Characteristics

The characteristics of our study participants by case–control status are presented in Table 1. We observed differences in several known factors that increase the risk of colorectal adenoma. Compared to the controls, cases were more likely to be older, male, have a higher BMI and WHR, and to consume more energy and saturated fat. For other factors, the cases and controls did not differ considerably.

### 3.2. Association between ω-3 PUFAs and Colorectal Adenomas

The results of the adjusted analyses showing the association between ω-3 PUFA consumption and colorectal adenomas are shown in Table 2. SC ω-3 PUFAs were the largest contributors to total ω-3 PUFA intake in this study population. The OR for those in the highest (≥1.80 g/day) relative to the lowest tertiles of the total ω-3 PUFA consumption was 0.49 (95% CI 0.23–1.01; p_trend_ = 0.06). A similar, but stronger and significant, association between SC ω-3 PUFAs and colorectal adenomas was observed (highest vs. lowest tertile OR = 0.45; 95 % CI = 0.21–0.97; p_trend_ = 0.04). The trend of an inverse association was not found for LC ω-3 PUFAs (p_trend_ = 0.22).

### 3.3. Association between Gut Microbiota Abundance and Colorectal Adenomas

Overall, we identified 37 bacteria at the genus level. The results of the differential abundance analysis for eighteen of the top-contributing bacteria and their association with colorectal adenomas are presented in Table 3. The abundance of *Sphingomonas* and *Marinomonas* at the genus level were significantly higher in the cases, while the abundances of *Sutterella* and *Parabacteroides* were significantly lower among the cases than among the controls, with FDR-adjusted *p*-values of < 0.05. There are statistically significant positive associations with colorectal adenomas per one-unit increment in the abundance of *Sphingomonas* (OR = 2.17; 95% CI = 1.31–3.57) and *Ralstonia* (OR = 1.19; 95% CI = 1.02–1.38), but an inverse association with the abundance of *Pseudoalteromonas* (OR = 0.58; 95% CI = 0.34–1.00). We did not observe statistically significant associations between bacterial diversity measures and colorectal adenomas.

### 3.4. Interactions between ω-3 PUFA and Gut Microbiota on Colorectal Adenoma

The associations between SC ω-3 PUFA and colorectal adenomas as stratified by the gut microbiota diversity measures and specific bacteria abundances are displayed in Table 4. We observed that the SC ω-3 PUFA–adenoma association differed in the bacteria evenness strata (*p*-_interaction_ < 0.05). Among the subjects who had higher levels of bacteria evenness, the risk of adenomas in relation to the consumption of higher levels of SC ω-3 PUFAs was greatly reduced (OR = 0.21; 95 % CI = 0.09–0.50). In contrast, the corresponding ω-3 PUFA–adenoma association among the lower bacteria evenness subgroup was increased to be above the null (OR = 1.10; 95 % CI = 0.50–2.43). In another comparison analysis, relative to those with both a low level of gut microbiota and a low intake of SC ω-3 PUFAs, adenoma risk tended to be the highest among those with both a high abundance of microbiota and a low intake of PUFAs. We found no significant differences in the SC ω-3 PUFA–adenoma association caused by bacteria diversity and richness nor by the abundance of six specific bacteria (*Sphingomonas, Marinomonas, Sutterella, Parabacteroides, Ralstonia, and Pseudoalteromonas*).

### 3.5. Association between BAs Concentration and Colorectal Adenomas

We further evaluated the association between colonic mucosal metabolites and adenomas in a subset of 25 cases and 25 controls. We identified 305 metabolites, 38 of which were significantly different between the cases and controls above a two-fold threshold (data not shown). A significant number of 11 microbial metabolites (BAs) were among these differential metabolites; two were primary BAs and nine were secondary BAs. The differential analysis and the association between these 11 BAs and colorectal adenomas are shown in Table 5. There was a positive association with colorectal adenoma per one-unit increment in the concentration of chenodeoxycholate (OR = 5.12; 95% CI = 1.17–22.44), taurochenodeoxycholate (OR = 4.45; 95% CI = 1.47–13.49), Taurocholate (OR = 2.80; 95% CI = 1.13–6.93), and taurodeoxycholate (OR = 3.68; 95% CI = 1.25–10.82).

### 3.6. Associations between BAs, Gut Microbiota, and Adenomas in the Subset of Samples with Metabolomics Data

**Figure 1:**
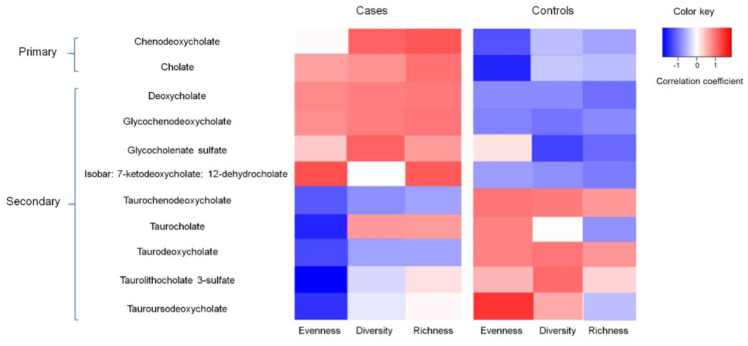
Correlation between secondary bile acid concentration and gut bacterial evenness, diversity, and richness among cases and controls, Diet and Health Study V, 2008–2010 (subset *n* = 50).

The correlation between BA concentration and gut microbiota diversity measures are shown in Figure 1. The correlation coefficients between the 11 BAs and bacterial diversity measures (evenness, diversity, and richness) among the cases were generally opposite to those among the controls. For example, the concentration of the primary BA chenodeoxycholate was positively correlated with bacterial diversity among the cases (r = 0.12, *p* = 0.06); however, it tended to be negatively correlated with bacterial diversity among the controls (r = −0.16, *p* = 0.22). The concentrations of 7-ketodeoxycholate and 12-dehydrocholate were consistently higher (fold changes > 2) among the subgroup of controls with high levels of bacterial richness and diversity as well as a high abundance of *Marinomonas, Parabacteroides* and low SC ω-3 PUFA intake (Supplemental Table S1).

### 3.7. Discussion

In this case–control study of 217 patients with adenomas and 218 adenoma-free controls, we found that SC ω-3 PUFAs were associated with a 47–55% reduced risk of developing colorectal adenomas. We also observed that the abundances of three specific gut bacteria and the concentrations of one primary and three secondary BAs measured in normal colonic mucosa tissue were associated with an increased risk of developing colorectal adenomas. Most surprisingly, we found that evenness, an estimator of the gut’s overall bacterial composition, may significantly modify the association between SC ω-3 PUFAs and colorectal adenomas. The risk reduction effect of SC ω-3 PUFAs was significantly more pronounced among those with a higher level of bacterial evenness, but not among those with lower bacterial evenness. Our findings also suggest that concentrations of specific BAs were correlated with gut bacterial composition and ω-3 PUFAs, with the correlation pattern being significantly different according to case–control status.

Our results for the non-significant association between LC ω-3 PUFA intake and colorectal adenomas are consistent with two previous epidemiologic findings [34,35]. The potential explanation for such discrepant results compared to experimental evidence may reflect low LC ω-3 PUFA intake in our study population. Furthermore, this could explain the inconsistent reports observed by other studies on ω-3 PUFA subtypes. As such, it could be that SC ω-3 PUFAs, especially ALAs, which are considered essential fatty acids, may be more relevant. Our finding of a direct risk reduction effect of SC ω-3 PUFAs (found mainly in seeds, nuts, and leafy green vegetables) are consistent with the accumulating laboratory evidence demonstrating the anti-inflammatory and anti-neoplastic activity of SC ω-3 PUFAs. In experimental models, it has been shown that SC ω-3 PUFAs decrease inflammatory eicosanoids and induce a cytotoxic environment within the cell by increasing levels of lipid peroxidation as well as by inducing apoptosis and reducing tumor cell growth [3,36,37]. Studying ω-3 PUFAs by subtype instead of as a whole may provide deeper insight into their distinguished role in CRC etiology, which may facilitate more precise nutritional prevention strategies.

The mucosal adherent microbial community composition has been suggested to play a role in the development of colorectal adenomas [12]. However, the contribution of specific bacterial signatures is not yet well-elucidated. Shen et al. and Sanapareddy et al. reported a higher proportion of *Proteobacteria* and lower abundance of *Bacteroidetes* in rectal mucosal biopsies of adenoma cases compared to non-adenoma controls [11,24]. Similar to these studies, of the top-contributing specific bacteria identified in our current study, six of eighteen belong to the phylum *Proteobacteria* and were shown to be positively associated with colorectal adenomas. Although not much is known about the clinical epidemiology and pathogenicity of these bacteria in humans, these bacteria could contribute to colorectal adenoma and cancer risk through changes in the local gut environment, such as altered gut pH, microbial metabolites, and local inflammation, which create different conditions for bacterial homeostasis/dysbiosis [11].

Analysis of the interaction between ω-3 PUFAs, the gut microbiota, and colorectal adenoma risk revealed the modifying role of gut bacterial evenness. A high intake of SC ω-3 PUFAs was significantly associated with a reduced risk of developing colorectal adenomas among a subgroup of subjects with high bacteria evenness, but not among those with low bacterial evenness, suggesting that the overall distribution of the bacterial community could be crucial for ω-3 PUFA metabolism and colorectal carcinogenesis. Several potential mechanisms support this important finding on the modification effect of bacteria evenness on the association between ω-3 PUFA intake and colorectal adenomas. A key aspect to consider is the role of ω-3 PUFAs and the resident gut microbiota in inflammation and immune regulation. It is believed that the commensal gut bacteria maintain a symbiotic interaction with the mucosal immune system and are in a state of tolerance under normal/healthy conditions. Disruption of the symbiotic environment may trigger chronic inflammation through increased mucosal permeability, bacterial translocation, and the increased activation of components of the innate and adaptive immune system [12,13,38]. Evenness could be regarded as a measure of overall gut bacterial distribution (how evenly the individuals in the community are distributed) [14]. Previous studies have observed that a higher level of evenness is associated with a stable state of tolerance between the microbial community and the gut immune system [39] and less chronic inflammation. Therefore, it is biologically plausible that ω-3 PUFAs would have a stronger impact on reducing the risk of developing colorectal adenomas for subjects with higher gut bacterial evenness (more stable and less inflamed environment). It is also possible that ω-3 PUFAs interact with the gut bacteria community by promoting the growth of specific bacteria. Previous experimental studies showed that dietary ω-3 PUFAs can reduce the growth of *Enterobacteria*; support the growth of *Bifidobacteria*, *Roseburia,* and *Lactobacillus*; and positively regulate the ratio of Firmicutes and Bacteroidetes (F/B) ratio [8,40]. The F/B ratio has been found to be significantly increased in subjects with metabolic diseases [40]. ω-3 PUFAs were also observed to have a strong association with gut bacterial composition diversity [41]. We found that our healthy controls with a higher ω-3 PUFA intake were more likely to have a low abundance of *Ralstonia* (which positively associated with colorectal adenoma in our samples). However, in general, we did not observe a significant association between ω-3 PUFA intake and gut microbiota composition, which is consistent with findings from a recent randomized trial [8]. Further research is needed to replicate our findings and to explicate the interaction between ω-3 PUFAs and the gut microbiota community (both overall distribution and specific bacteria) in colorectal carcinogenesis.

In a subset of subjects with mucosal metabolomics data, we found a positive association between the concentrations of specific BAs (particularly taurine-conjugated secondary BAs) and colorectal adenomas. Our findings are also consistent with previous laboratory studies [42,43,44]. It is also interesting to find that all the differential bile acids were higher in the cases than in the controls. There is a possibility that the higher level of saturated fat intake and BMI of the cases (Supplemental Table S2) lead to the upregulation of total cholesterol/bile acid levels in the liver or colon. Secondary BAs are an important class of metabolites produced by colonic bacterial flora [45]. An aberrant increased concentration of primary and secondary BAs may contribute to colonic mucosal epithelial damage and may lead to increased ROS, DNA damage, genomic instability, and tumor growth [46]. However, only a few human studies with sample sizes < 25 [19,47,48,49] have evaluated secondary BAs in relation to colorectal adenomas/cancer. Moreover, the secondary BAs in these studies were predominately measured in serum and fecal samples. The BA composition in the large intestine, plasma, and feces is significantly different [50]. To understand the precise role of BAs in carcinogenesis, information on BA composition at the normal mucosal tissue level, as reflected in our present findings, is essential.

Although we were unable to perform an interaction analysis to examine the modification effect of BAs on the ω-3 PUFA intake–adenoma association due to the limited sample size, we observed a negative correlation between the SC ω-3 PUFA and BA concentrations measured in normal mucosal tissue. There is a hypothesis that ω-3 PUFAs may promote bile discharge and increase the quantity of BAs that escape enterohepatic recirculation [19] without affecting BA pool size or cholesterol synthesis [18]. Previous studies provided evidence for this hypothesis by observing a positive correlation between ω-3 PUFAs and the circulating BA concentration (measured in serum and feces) [18,51]. Our observation of a negative correlation of ω-3 PUFAs and non-circulating tissue-level BA concentrations indirectly supports the above biological hypothesis (given the stable BA pool size) and provides novel biological evidence from tissue-level differences for the beneficial effect of ω-3 PUFAs in protecting against colorectal carcinogenesis. We also report that the correlations between the BA concentration and bacteria diversity measures were significantly different based on adenoma status. In particular, we observed a positive correlation between several secondary BAs and bacterial diversity and richness among the cases, which may suggest that the interplay between microbiota and microbial metabolites is important in the development of colorectal adenomas. Our findings are consistent with those from a previous report, which suggested that adenoma patients had an altered microbiota profile and that this signature could lead an increased level of primary and secondary BA production [52].

This study has several limitations. First, our findings depend on self-reported ω-3 PUFA intake determined via FFQ. This could result in potential misclassification and, for the case–control study, recall bias. However, our FFQ was validated to represent dietary intake habits for the past 5–10 years, which is also the standard for nutritional epidemiology research. In addition, we observed a strong positive association between ω-3 PUFA-related tissue-cased metabolites such as docosapentaenoate with colorectal adenomas (high vs. low concentration OR = 2.28; 95% CI = 1.00–5.19), which may support the results of the questionnaire-based ω-3-PUFA measurements well. Furthermore, all the FFQs and other study questionnaires were completed within 12 weeks of colonoscopy and diagnoses to minimize bias. Similarly, several features of ω-3 PUFA intake, such as harvesting, storage, processing, and cooking methods, may also influence the accuracy of reporting due to the PUFA content measured in the US Department of Agriculture database potentially differing from our participants’ actual consumption [53]. Second, although we evaluated and adjusted for a range of potentially confounding factors, including the overall bacterial composition diversity measures, residual confounding bias by unknown factors related to the indication of ω-3 PUFA intake could not be ruled out. Third, the case–control nature of our study design does not allow us to parse correlation versus causation between ω-3 PUFA intake, gut microbiota, BAs concentration, and adenoma status, and the gut microbiome may be altered by adenoma status. However, our findings provide important insights into the mechanisms that may be driving adenoma development and have the potential to inform future studies in animal models to evaluate those mechanisms. Fourth, we only measured the microbiota in the mucosal tissues, and fecal samples were not available to investigate the correlation between the tissue and fecal samples, and we were also not able to compare our results to most of the other studies in this domain that based on fecal samples only. Fifth, although we have performed strict quality control processes, we cannot rule out the chance that bacterial contamination during sample collection may have influenced our findings. Lastly, we were only able to measure mucosal tissue-level BAs among a subgroup of ~50 subjects from the same geographic region (North Carolina, USA) because of the extremely expensive cost of global metabolomic analysis. Larger study populations in various regions would be needed to provide sufficient power to examine the association between BAs and colorectal adenomas and to explore the potential modifying/mediating role of BAs on the association between ω-3 PUFAs and colorectal adenomas.

To the best of our knowledge, the current study is the first to examine the potential modifying effect of the gut microbiota on the association between ω-3 PUFA intake and the risk of developing colorectal adenomas as well as the first to identify the correlation pattern between ω-3 PUFAs and bacteria composition with the colonic mucosal tissue-level BA concentration. The questionnaires were administered within months of colonoscopy, which was likely to reduce the possibility of dietary changes due to adenoma status and the impact of recall bias when completing the FFQ. Strengths of this study also include the consecutive recruitment of study participants, the use of colonoscopy and pathological evaluations of polyps to classify the participants to adenoma status, and the extensive data on established and potential risk factors for colon adenomas. The risk factors (e.g., age, sex, and BMI) identified in Table 1 were consistent with previous findings [54]. The depth of microbiota 16S rRNA gene sequencing and the larger sample size in this study provides better coverage and a better understanding of the overall composition of the microbial communities and BA metabolism. Differences between sequencing platforms may also explain the reason why we did not find a positive association between richness and adenomas, which was the case in our previous publication [10]. We also had a larger sample size and used the Illumina platform instead of pyrosequencing [10] in the present study.

## 4. Conclusions

In summary, we found significant associations between SC ω-3 PUFA consumption, specific gut bacteria abundances, and colonic tissue-level BA concentrations that had a direct influence on the occurrence of colorectal adenomas. We also identified the interactions among all three of these factors, which suggests complex interplay between ω-3 PUFAs, the microbiota, and BAs that contributes to the development of colorectal adenomas. Our results imply that improved ω-3 PUFA intake and/or alterations in the gut microbial environment may become a potential effective risk reduction strategy for colorectal cancer prevention. Furthermore, our observation of the interaction between ω-3 PUFA intake and bacteria evenness and the negative correlation between ω-3 PUFA intake and tissue-level BA concentration requires replication and further investigation into the underlying biological mechanism. This information could help to identify sub-groups of population that may be more susceptible to the effects of increasing ω-3 PUFA intake and may yield more precise preventive and therapeutic interventions to improve outcomes for patients with colorectal adenoma and/or cancer.

## Figures and Tables

**Table 1:** Descriptive characteristics of the study participants, Diet and Health Study V, 2008–2010 (*n* = 435).

Selected Characteristics	Cases (*n* = 217)	Controls (*n* = 218)	*p*-Value
Age (years), mean (SD)	56.3 (6.9)	55.2 (6.3)	0.19
Male (%)	52.3	44.2	0.09
White (%)	81.0	85.3	0.31
Family history of colorectal cancer in first-degree relative (%)	3.4	4.6	0.54
Regular (≥once/week) NSAID use (%)	56.9	50.8	0.23
Total energy intake (kcal/day), mean (SD)	1992.4 (847.5)	1880.8 (726.0)	0.16
Total ω-3 polyunsaturated fat intake (g/day), mean (SD)	1.7 (1.0)	1.6 (0.7)	0.37
Total ω-6 polyunsaturated fat intake (g/day), mean (SD)	15.5 (9.3)	14.8 (6.9)	0.42
Total saturated fat intake (g/day), mean (SD)	24.1 (13.0)	22.2 (10.1)	0.10
Total vegetables intake (servings/day), mean (SD)	4.5 (2.6)	4.4 (2.4)	0.70
Total fruit intake (servings/day), mean (SD)	2.8 (1.9)	2.8 (1.8)	0.90
Red meat (oz/day), mean (SD)	1.5 (1.2)	1.4 (1.2)	0.30
Dietary fiber intake (g/day), mean (SD)	20.3 (9.8)	20.0 (9.3)	0.73
Total calcium intake (mg/day), mean (SD)	882.2 (421.0)	836.3 (401.2)	0.27
Total folate intake (mcg/day), mean (SD)	418.7 (247.0)	425.5 (244.9)	0.61
Total vitamin E intake (mg/day), mean (SD)	11.1 (7.4)	10.5 (4.6)	0.37
Ever Smoked (%)	42.5	45.7	0.52
Alcohol intake, mean (SD)	12.4 (17.1)	11.0 (18.3)	0.40
Body mass index (kg/m^2^), mean (SD)	28.1 (5.7)	27.1 (5.7)	0.07
Waist-to-hip ratio, mean (SD)	0.93 (0.1)	0.91 (0.1)	0.01
Distal adenoma (%), mean (SD)	65.2	N.A.	N.A.
Adenoma size (cm), mean (SD)	5.5 (5.0)	N.A.	N.A.
Bacteria diversity, mean (SD)	8.6 (3.6)	8.3 (3.8)	0.42
Bacteria evenness, mean (SD)	0.7 (0.2)	0.7 (0.3)	0.24
Bacteria richness, mean (SD)	6.8 (2.6)	6.6 (2.8)	0.41

Abbreviations: SD, standard deviation; NSAID, nonsteroidal anti-inflammatory drug. N.A., not applicable.

**Table 2:** Association between ω-3 polyunsaturated fatty acid intake and colorectal adenomas, Diet and Health Study V, 2008–2010 (*n* = 435).

Dietary Factors	Cases/Controls, *n*	Adjusted OR (95% CI) ^a^
Total ω-3 PUFA intake, g/day		
By tertiles		
<1.25	63/64	1.00 (Ref)
1.25– < 1.80	72/65	0.80 (0.45–1.42)
≥1.80	68/65	0.49 (0.23–1.01)
*By median*		
<1.47	94/96	1.00 (Ref)
≥1.47	109/98	0.72 (0.41–1.24)
P_trend_		0.06
Short-chain ω-3 PUFA intake, g/day		
By tertiles		
<1.16	66/64	1.00 (Ref)
1.16– < 1.64	67/65	0.69 (0.39–1.22)
≥1.64	70/65	0.45 (0.21–0.97)
*By median*		
<1.37	100/95	1.00 (Ref)
≥1.37	103/99	0.53 (0.30–0.92)
P_trend_		0.04
Long-chain ω-3 PUFA intake, g/day		
By tertiles		
<0.07	56/52	1.00 (Ref)
0.07– < 0.12	56/66	0.63 (0.35–1.10)
≥0.12	91/76	0.89 (0.51–1.54)
*By median*		
<0.09	81/91	1.00 (Ref)
≥0.09	120/105	1.17 (0.74–1.87)
P_trend_		0.22

Abbreviations: PUFA, polyunsaturated fatty acid; OR, odds ratio; CI: confidence interval. ^a^ Adjusted multi-variables: age, sex, race, BMI, total energy intake, saturated fat intake, vitamin E intake, bacteria diversity, evenness, and richness.

**Table 3:** Associations between gut microbiota and colorectal adenomas, Diet and Health Study V, 2008–2010 (*n* = 435).

Characteristics	Controls/Cases, *n*	FC	FDR	Crude OR (95% CI)	Multivariable-Adjusted OR (95% CI) ^a^
Bacteria taxa (Phylum; genus)					
Proteobacteria; *Sphingomonas*	203/208	0.0642	2.12 × 10^−13^	2.18 (1.34–3.55)	2.17 (1.31–3.57)
Proteobacteria; *Marinomonas*	203/208	0.2053	3.24 × 10^−6^	1.18 (0.85–1.64)	1.14 (0.81–1.60)
Proteobacteria; *Sutterella*	203/208	1.9198	0.0326	0.78 (0.55–1.10)	0.80 (0.56–1.14)
Bacteroidetes; *Parabacteroides*	203/208	1.7654	0.0326	0.91 (0.65–1.28)	0.94 (0.66–1.33)
Bacteroidetes; *Bacteroides*	203/208	1.4240	0.0676	0.98 (0.84–1.14)	1.02 (0.87–1.20)
Firmicutes; *Streptococcus*	203/208	0.5705	0.0676	0.98 (0.79–1.22)	0.94 (0.75–1.18)
Proteobacteria; *Pseudoalteromonas*	203/208	0.5809	0.1577	0.58 (0.34–0.99)	0.58 (0.34–1.00)
Firmicutes; Blautia	203/208	0.7481	0.1874	1.14 (0.93–1.41)	1.17 (0.94–1.47)
Firmicutes; *Roseburia*	203/208	1.3751	0.2281	0.98 (0.75–1.27)	0.96 (0.73–1.28)
Firmicutes; *Phascolarctobacterium*	203/208	1.4854	0.2281	0.92 (0.67–1.25)	1.02 (0.73–1.43)
Proteobacteria; *Ralstonia*	203/208	0.5593	0.2281	1.20 (1.04–1.39)	1.19 (1.02–1.38)
Proteobacteria; *Bilophila*	203/208	1.2571	0.3822	1.20 (0.76–1.91)	1.39 (0.84–2.30)
Actinobacteria; *Collinsella*	203/208	1.1777	0.6344	1.02 (0.88–1.18)	0.99 (0.85–1.16)
Actinobacteria; *Propionibacterium*	203/208	1.1727	0.7359	1.04 (0.78–1.41)	1.01 (0.73–1.38)
Actinobacteria; *Bifidobacterium*	203/208	1.0527	0.9706	1.08 (0.78–1.51)	0.99 (0.70–1.40)
Firmicutes; *Coprococcus*	203/208	0.9833	0.9706	1.03 (0.84–1.26)	1.06 (0.85–1.31)
Firmicutes; *Ruminococcus*	203/208	0.9893	0.9706	1.12 (0.89–1.41)	1.14 (0.89–1.44)
Firmicutes; *Dorea*	203/208	0.9936	0.9706	1.08 (0.92–1.28)	1.09 (0.91–1.30)
Bacteria Overall Composition Measurements					
Richness	203/208	--	--	1.03 (0.96–1.11)	1.04 (0.96–1.13)
Evenness	203/208	--	--	1.61 (0.73–3.54)	1.91 (0.83–4.39)
Diversity	203/208	--	--	1.02 (0.97–1.08)	1.04 (0.98–1.10)

Abbreviations: OR, odds ratio; CI: confidence interval; FC: fold changes; FDR, false discovery rate. ^a^: adjusted by age, sex, and race.

**Table 4:** Adjusted multi-variable ^a^ interaction analysis of short-chain ω-3 polyunsaturated fatty acid intake and gut microbiota on colorectal adenomas, Diet and Health Study V, 2008–2010 (*n* = 435).

Bacteria Characteristics	Short-Chain ω-3 PUFA Intake, g/Day	Cases (*n*)	Controls (*n*)	Single-Referenced ORs (95% CIs)	Stratified ORs (95% CIs)	*p*-_interaction_
**Bacteria richness**						
<Median (7.38)	<Median (1.37)	46	44	1.00	1.00	
	≥Median (1.37)	43	45	0.52 (0.26, 1.06)	0.45 (0.20, 1.02)	
≥Median (7.38)	<Median (1.37)	54	43	1.52 (0.69, 3.34)	1.00	
	≥Median (1.37)	51	47	0.79 (0.33, 1.87)	0.58 (0.26, 1.27)	0.93
**Bacteria evenness**						
<Median (0.80)	<Median (1.37)	47	52	1.00	1.00	
	≥Median (1.37)	49	37	0.84 (0.40, 1.75)	1.10 (0.50, 2.43)	
≥Median (0.80)	<Median (1.37)	53	35	1.63 (0.77, 3.45)	1.00	
	≥Median (1.37)	45	55	0.58 (0.25, 1.30)	0.21 (0.09, 0.50)	0.03
**Bacteria diversity**						
<Median (9.33)	<Median (1.37)	47	42	1.00	1.00	
	≥Median (1.37)	44	50	0.46 (0.23, 0.94)	0.32 (0.14, 0.75)	
≥Median (9.33)	<Median (1.37)	53	45	1.09 (0.49, 2.38)	1.00	
	≥Median (1.37)	50	42	0.65 (0.27, 1.59)	0.69 (0.32, 1.48)	0.44
***Sphingomonas***						
<Median (0.00)	<Median (1.37)	65	49	1.00	1.00	
	≥Median (1.37)	50	57	0.40 (0.21, 0.78)	0.40 (0.19, 0.85)	
≥ Median (0.00)	<Median (1.37)	35	38	0.68 (0.36, 1.27)	1.00	
	≥Median (1.37)	44	35	0.55 (0.27, 1.12)	0.51 (0.18, 1.45)	0.32
***Marinomonas***						
<Median (0.01)	<Median (1.37)	36	40	1.00	1.00	
	≥Median (1.37)	35	48	0.49 (0.23, 1.02)	0.32 (0.13, 0.81)	
≥Median (0.01)	<Median (1.37)	64	47	1.69 (0.87, 3.28)	1.00	
	≥Median (1.37)	59	44	1.01 (0.47, 2.14)	0.76 (0.35, 1.68)	0.08
***Sutterella***						
<Median (0.04)	<Median (1.37)	53	43	1.00	1.00	
	≥Median (1.37)	47	48	0.43 (0.21, 0.89)	0.55 (0.24, 1.26)	
≥Median (0.04)	<Median (1.37)	47	44	0.81 (0.43, 1.55)	1.00	
	≥Median (1.37)	47	44	0.51 (0.24, 1.08)	0.43 (0.18, 1.01)	0.53
***Parabacteroides***						
<Median (0.30)	<Median (1.37)	54	42	1.00	1.00	
	≥Median (1.37)	49	48	0.49 (0.25, 0.96)	0.54 (0.24, 1.20)	
≥Median (0.30)	<Median (1.37)	46	45	0.82 (0.43, 1.55)	1.00	
	≥Median (1.37)	45	44	0.40 (0.22, 1.00)	0.48 (0.20, 1.15)	0.49
***Pseudoalteromonas***						
<Median (0.04)	<Median (1.37)	47	50	1.00	1.00	
	≥Median (1.37)	49	41	0.69 (0.34, 1.41)	0.74 (0.33, 1.65)	
≥Median (0.04)	<Median (1.37)	53	37	1.34 (0.72, 2.50)	1.00	
	≥Median (1.37)	45	51	0.54 (0.27, 1.10)	0.38 (0.17, 0.85)	0.44
***Ralstonia***						
<Median (0.00)	<Median (1.37)	45	43	1.00	1.00	
	≥Median (1.37)	34	44	0.49 (0.23, 1.03)	0.66 (0.28, 1.56)	
≥ Median (0.00)	<Median (1.37)	55	44	1.17 (0.62, 2.19)	1.00	
	≥Median (1.37)	60	48	0.64 (0.31, 1.30)	0.38 (0.16, 0.87)	0.49

Abbreviations: PUFA, polyunsaturated fatty acid; OR, odds ratio; CI: confidence interval. ^a^ Adjusted: age, sex, race, BMI, total energy intake, saturated fat intake, vitamin E intake, bacteria diversity, evenness, and richness.

**Table 5:** Associations between significant bile acid concentration and colorectal adenomas, Diet and Health Study V, 2008–2010 (subset *n* = 50).

Microbial Metabolites/Bile Acids	Cases/Controls, *n*	FC	FDR	OR (95% CI) ^a^
Cholate	23/11	3.5372	0.0005	1.06 (0.51–2.18)
Taurolithocholate 3-sulfate	15/3	5.0002	0.0005	N.A.
Taurocholate	20/12	6.5657	0.0007	2.80 (1.13–6.93)
Taurochenodeoxycholate	18/13	6.3328	0.0028	4.45 (1.47–13.49)
Taurodeoxycholate	17/11	8.3950	0.0038	3.68 (1.25–10.82)
Glycocholenate sulfate	10/1	3.3605	0.0042	N.A.
Tauroursodeoxycholate	11/2	3.5215	0.0072	N.A.
Chenodeoxycholate	13/9	19.883	0.0113	5.12 (1.17–22.44)
Isobar: 7-ketodeoxycholate; 12-dehydrocholate	13/5	2.4302	0.0144	1.34 (0.49–3.68)
Glycochenodeoxycholate	12/8	11.6478	0.0312	3.11 (0.89–10.90)
Deoxycholate	22/19	3.5998	0.0394	1.86 (0.93–3.73)
Glycocholate	15/11	6.7675	0.0886	1.86 (0.76–3.54)
Glycoursodeoxycholate	7/3	3.7757	0.0941	N.A.

Abbreviations: OR, odds ratio; CI: confidence interval; FC: fold changes; FDR, false discovery rate; N.A. not applicable because cell sizes were less than 5. ^a^ Crude analysis because of the limited sample size.

## Data Availability

The Diet and Health Study V resources are stored at UNC Chapel Hill. Information regarding data availability can be obtained by contacting the principal investigator of this study: Dr. Temitope O. Keku (tokeku@med.unc.edu).

## Supplementary Materials

The following supporting information can be downloaded at https://www.mdpi.com/article/10.3390/cancers14184443/s1: Table S1: Differential microbial metabolites (bile acids) by gut microbiota and short-chain ω-3 PUFA levels among controls; Table S2: Descriptive characteristics of study participants with microbial metabolite (bile acid) measurements, Diet and Health Study V, 2008–2010 (*n* = 50).
