# Supplementary material for: Omega-3 Polyunsaturated Fatty Acids, Gut Microbiota, Microbial Metabolites, and Risk of Colorectal Adenomas
Source: Cancers (Basel). 2022 Sep 13;14(18):4443. doi: 10.3390/cancers14184443 (PMC9496906; doi:10.3390/cancers14184443)
Supplement: Supplementary file 1 [file cancers-14-04443-s001.zip › cancers-1891194-supplementary.pdf]

Supplemental Table S1. Differential Microbial Metabolites (Bile Acids) by Gut Microbiota and Short-Chain ω -3 PUFA Levels Among Controls

Level	Differential Metabolites	Fold Change
Richness (low vs. high)	Glycocholate	2.0621
	7-ketodeoxycholate; 12-dehydrocholate	0.4238
Evenness (low vs. high)	Glycocholate	2.2711
Diversity (low vs. high)	7-ketodeoxycholate; 12-dehydrocholate	0.4867
Sphingomonas (low vs. high)	Chenodeoxycholate	2.2989
Marinomonas (low vs. high)	7-ketodeoxycholate; 12-dehydrocholate	0.4612
	Taurodeoxycholate	0.4652
Sutterella (low vs. high)	Cholate	2.3529
Parabacteroides (low vs. high)	7-ketodeoxycholate; 12-dehydrocholate	0.4081
Pseudoalteromonas (low vs. high)	None	N.A.
Ralstonia (low vs. high)	Chenodeoxycholate	2.0402
Short-chain omega-3 PUFA (low vs. high)	7-ketodeoxycholate; 12-dehydrocholate	2.1823

Supplemental Table S2. Descriptive Characteristics of Study Participants with Microbial Metabolites (Bile Acids) Measurements, Diet and Health Study V, 2008-2010 (N=50)

Selected Characteristics	Cases	Controls
Age (years), mean (SE)	54.4 (4.2)	57 (8.7)
Male (%)	40.9	64.0
White (%)	81.8	96.0
Family history of colorectal cancer in 1st degree relative (%)	3.4	4.6
Regular (≥ once/week) NSAID use (%)	65.0	57.1
Total energy intake (kcal/day), mean (SE)	1,771.5 (675.1)	1,815.9 (716.3)
Total ω-3 polyunsaturated fat intake (g/day), mean (SE)	1.7 (0.7)	1.6 (0.7)
Total ω-6 polyunsaturated fat intake (g/day), mean (SE)	15.2 (6.8)	13.8 (6.5)
Total saturated fat intake (g/day), mean (SE)	24.3 (11.5)	20.7 (8.9)
Total vegetables intake (servings/day), mean (SE)	3.9 (2.2)	4.5 (2.9)
Total fruit intake (servings/day), mean (SE)	2.2 (1.7)	3.0 (2.3)
Red meat (oz/day), mean (SE)	1.4 (0.8)	1.4 (1.5)
Dietary fiber intake (g/day), mean (SE)	18.2 (7.6)	20.6 (10.5)
Total calcium intake (mg/day), mean (SE)	852.6 (549.3)	753.6 (301.3)
Total folate intake (mcg/day), mean (SE)	367.2 (133.9)	412.8 (193.1)
Total vitamin E intake (mg/day), mean (SE)	10.0 (4.4)	10.1 (4.5)
Ever Smoked (%)	45.0	52.4
Alcohol intake, mean (SE)	10.8 (25.7)	9.7 (9.1)
Body mass index (kg/m^2), mean (SE)	30.0 (8.7)	26.8 (4.3)
Waist-to-hip ratio, mean (SE)	0.93 (0.1)	0.94 (0.1)
Distal adenoma (%), mean (SE)	N.A.	N.A.
Adenoma size (cm), mean (SE)	6.8 (3.9)	N.A.
Bacteria diversity, mean (SE)	6.9 (3.2)	6.6 (3.1)
Bacteria evenness, mean (SE)	0.7 (0.3)	0.7 (0.3)
Bacteria richness, mean (SE)	9.0 (7.0)	8.5 (6.8)

Abbreviations: SE, standard error; NSAID, nonsteroidal anti-inflammatory drug; N.A. not applicable.
